# Comparison of T-piece and pressure support ventilation as spontaneous breathing trials in critically ill patients: a systematic review and meta-analysis

**DOI:** 10.1186/s13054-020-2764-3

**Published:** 2020-02-26

**Authors:** Yuting Li, Hongxiang Li, Dong Zhang

**Affiliations:** Department of Intensive Care Unit, The First Hospital of Jilin University, Changchun, Jilin, 130021 China

**Keywords:** T-piece, Pressure support ventilation, Spontaneous breathing trials, Randomized controlled trials, Systematic review, Meta-analysis

## Abstract

**Background:**

The effect of alternative spontaneous breathing trial (SBT) techniques on extubation success and other clinically important outcomes is uncertain. A systematic review and meta-analysis was performed to clarify the preferable SBT (T-piece or pressure support ventilation [PSV]).

**Methods:**

We searched the PubMed, Cochrane, and Embase databases for randomized controlled trials (RCTs) from inception to the 31st of July 2019. We included RCTs involving adult patients (> 18 years) who underwent at least two different SBT methods. All authors reported our primary outcome of successful extubation rate and clearly compared PS versus T-piece with clinically relevant secondary outcomes (rate of reintubation, ICU and hospital length of stay, and ICU and hospital mortality). Results were expressed as odds ratio (OR) and mean difference (MD) with accompanying 95% confidence interval (CI).

**Results:**

Ten RCTs including 3165 patients were included. The results of this meta-analysis showed that there was no significant difference in the successful extubation rate between the T-piece group and PS group (odds ratio [OR] = 0.91; 95% CI, 0.78–1.07; *P* = 0.27; *I*^2^ = 79%). In addition, compared with the PS group, the T-piece group showed no significant difference in the rate of reintubation (odds ratio [OR] = 0.99; 95% CI, 0.78–1.26; *P* = 0.95; *I*^2^ = 5%), ICU mortality (odds ratio [OR] = 1.22; 95% CI, 0.83–1.80; *P* = 0.30; *I*^2^ = 0%), hospital mortality (odds ratio [OR] = 1.36; 95% CI, 0.99–1.87; *P* = 0.06; *I*^2^ = 19%), ICU length of stay (mean difference = − 0.10; 95% CI, − 0.59 to 0.39; *P* = 0.69; *I*^2^ = 13%), and hospital length of stay (mean difference = − 0.82;95% CI, − 2.2 to 0.55; *P* = 0.24; *I*^2^ = 0%).

**Conclusions:**

T-piece and PSV as SBTs are considered to have comparable predictive power of successful extubation in critically ill patients. The analysis of secondary outcomes also shows no significant difference in the rate of reintubation, ICU and hospital length of stay, and ICU and hospital mortality between the two groups. Further randomized controlled studies of SBTs are still required.

## Key messages

- T-piece and PSV as SBTs are considered to have comparable predictive power of successful extubation in critically ill patients.
- Further randomized controlled studies of SBTs are still required to confirm our results.

## Background

Mechanical ventilation is often required in patients with critical illness, but after recovery from the acute illness, several problems can impair the successful separation of the patient from the ventilator [1]. Weaning from mechanical ventilation is one of the most important and challenging problems for most intensive care unit (ICU) patients. It is well known that weaning failure is associated with longer use of mechanical ventilation, higher infection rate, longer ICU stay, longer hospital stay, and higher mortality rate [2]. A spontaneous breathing trial (SBT) is most often performed to assess the ability of a patient to sustain spontaneous breathing when extubated [3]. The most common modes of SBT are T-piece ventilation and pressure support ventilation (PSV), lasting between 30 min and 2 h [4–6].

Discontinuation of mechanical ventilation should be accomplished when the patient’s ability to breathe unassisted is identified. Both premature and delayed ventilator discontinuation are associated with significant morbidity. Daily spontaneous breathing trials (SBTs) are the current evidence-based standard of care in determining the time of ventilator discontinuation. When patients are ready to wean, the weaning process should be initiated with the first SBT as soon as possible. Nevertheless, about 15–30% of the patients will be re-intubated even if they are able to tolerate (or pass) the SBT [7].

A recent meta-analysis suggested that patients undergoing PS (vs T-piece) SBTs appear to be 6% more likely to be extubated successfully and, if the results of an outlier trial are excluded, 6% more likely to pass an SBT [8]. Another meta-analysis found that PSV might be superior to T-piece with regard to weaning success for simple-to-wean subjects. For the prolonged-weaning subgroup, however, T-piece was associated with a shorter weaning duration [9]. A latest large-scale multicenter randomized controlled trial found that an SBT consisting of 30 min of PSV, compared with 2 h of T-piece ventilation, led to significantly higher rates of successful extubation [10]. Moreover, the latest American Thoracic Society guidelines for weaning recommend PSV SBTs with moderate-quality evidence [11]. Thus, further research is needed to determine the best approach for SBTs.

In this study, we conducted a meta-analysis, which extracted results from published randomized controlled trials (RCTs) to evaluate the effectiveness and safety of two strategies, a T-piece and PSV, for weaning adult patients with respiratory failure that required mechanical ventilation, measuring extubation success and other clinically important outcomes.

## Methods

This systematic review and meta-analysis is reported according to the Preferred Reporting Items for Systematic Reviews and Meta-Analyses (PRISMA) guidelines [12]. Ethical approval was not necessary for this study because it was a review of the published literature.

### Search strategy

**Fig. 1:**
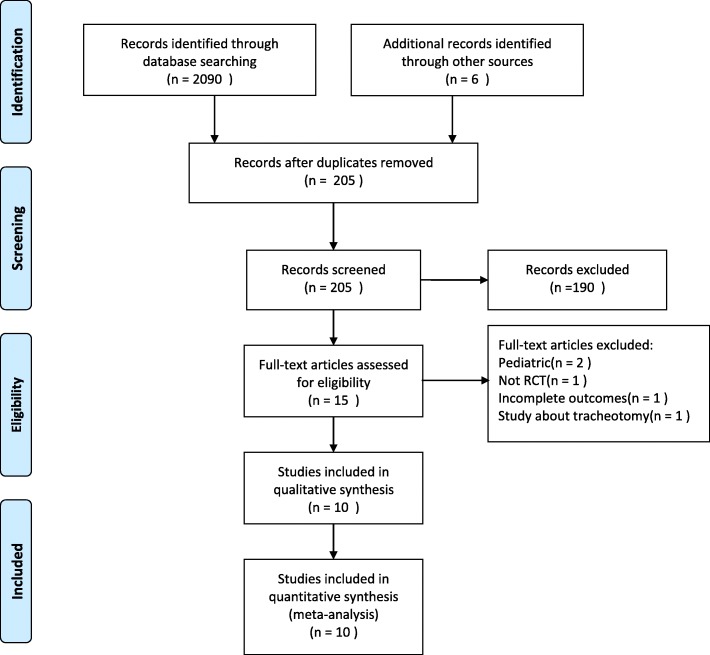
Flow chart of literature selection

We searched the PubMed, Cochrane, and Embase databases for RCTs from inception to the 31st of July 2019 using the following search terms: spontaneous breathing trial, T-piece, T-tube, pressure support ventilation, pressure support, weaning, ventilator weaning, mechanical ventilation. The search was slightly adjusted according to the requirements of the different databases. The authors’ personal files and reference lists of relevant review articles were also reviewed. The flow chart of the search strategies is summarized in Fig. 1.

### Types of outcome measures

The primary outcome was successful extubation rate, and successful extubation was defined as remaining free of invasive mechanical ventilation 72 h after the first SBT [10].

Secondary outcomes were rate of reintubation among patients who were extubated after the SBT, ICU, and hospital length of stay, and ICU and hospital mortality. Weighted means were calculated based on the number of patients in each study.

### Study selection

The inclusion criteria were as follows: (1) randomized controlled trials; (2) adult patients (> 18 years) who underwent at least two different SBT methods; (3) all authors reported our primary outcome of successful extubation rate; (4) clearly comparing PS versus T-piece with clinically relevant secondary outcomes. We excluded nonrandomized controlled trials and studies without clear comparisons of the outcomes. In addition, we excluded studies evaluating SBT methods in patients with tracheotomy and in patients receiving noninvasive ventilation.

### Quality assessment

**Fig. 2:**
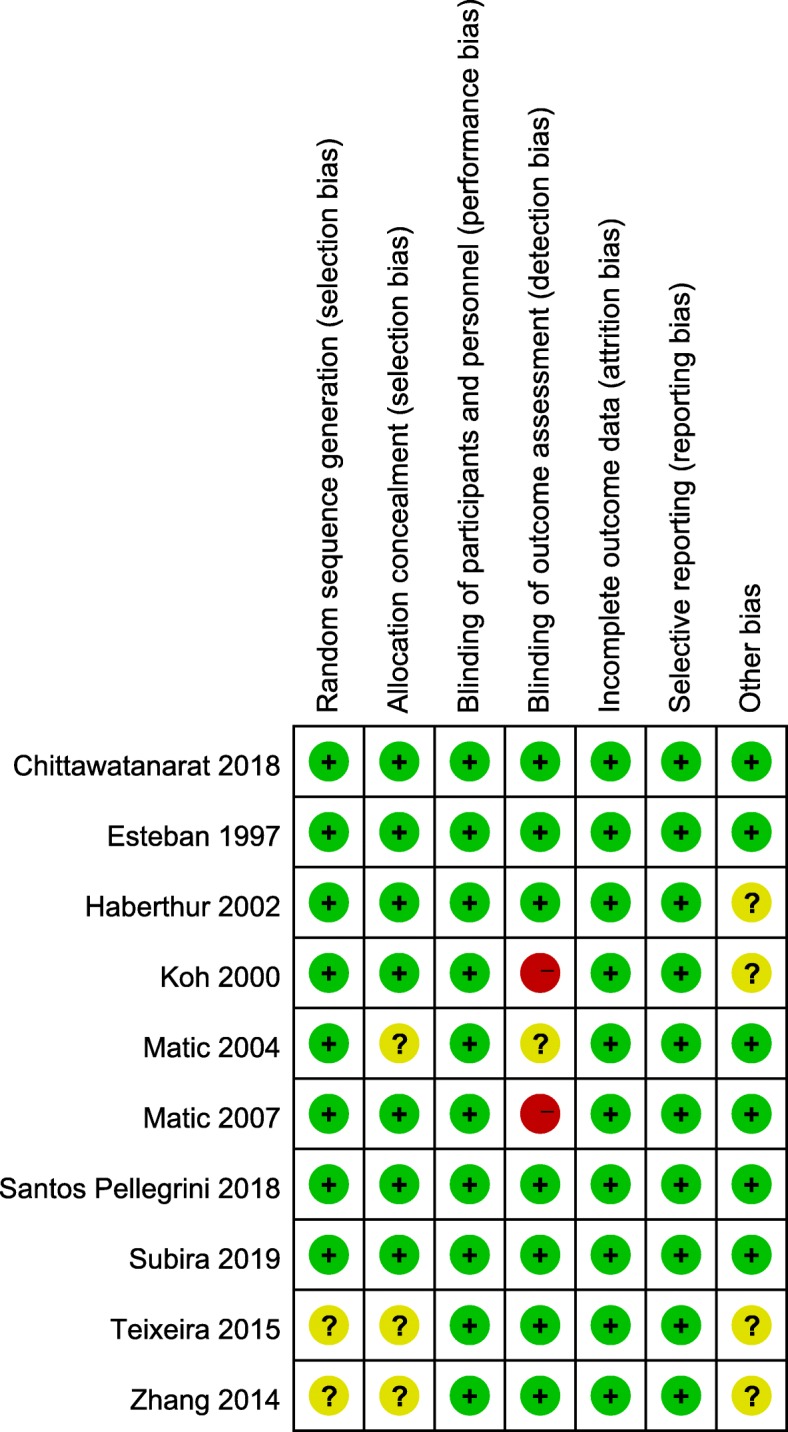
Risk of bias summary: review authors’ judgements about each risk of bias item for each included study

**Fig. 3:**
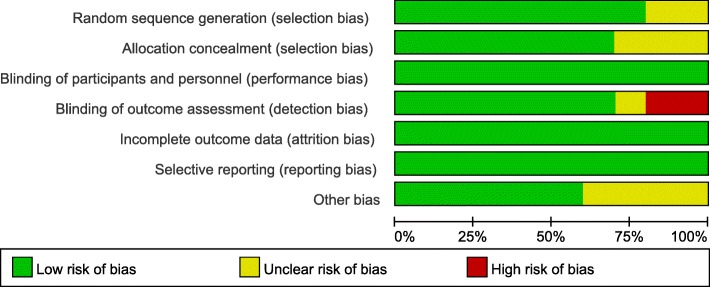
Risk of bias graph: review authors’ judgements about each risk of bias item presented as percentages across all included studies

Two reviewers (YL and HL) independently performed quality assessment using the Cochrane Collaboration’s tool for assessing risk of bias [13]. The specific elements were adequacy of the methods used to minimize bias through: (1) randomization sequence (selection bias), (2) allocation concealment (selection bias), (3) blinding of study personnel and participants (performance bias), (4) blinding of outcome assessors (performance bias), (5) complete reporting of data without arbitrarily excluded patients and with low to minimal loss to follow-up (attrition bias), (6) selective reporting bias, and (7) other sources of bias. Satisfactory performance, unclear performance, and unsatisfactory performance of each domain from the tool is denoted by green, yellow, and red colors respectively. The risk of bias summary is presented in Fig. 2; the risk of bias graph is presented in Fig. 3.

### Statistical analysis

Statistical analyses were performed using Review Manager Version 5.3 (RevMan, The Cochrane Collaboration, Oxford, UK). Risk ratio (RR) with 95% confidence intervals (CI) was calculated for dichotomous variables. As to the continuous variables, mean difference (MD) and 95% CI was estimated as the effect result. A random-effects model was used to pool studies with significant heterogeneity, as determined by the chi-squared test (*P* < 0.10) and inconsistency index (*I*^2^ ≥ 50%) [14]. Some of the selected continuous variables were represented by the median (interquartile range). We calculated their mean and standard deviation according to the sample size with a calculator [15], and then performed meta-analysis. A *P* value < 0.05 was set as the threshold of statistical significance.

## Result

### Study characteristics

The search strategy identified 2090 studies, and the data were from 10 RCTs comprising 3165 patients (Table 1) [10, 16–24]. The characteristics of the included studies are shown in Table 1. A total of 10 eligible studies were published between 1997 and 2019. Among these studies, 2 studies were conducted in Spain, 2 studies were conducted in Croatia, 2 studies were conducted in Brazil, 1 study was conducted in Korea, 1 study was conducted in Switzerland, 1 study was conducted in Thailand, and 1 study was conducted in China. Of these studies, three were multi-center studies [10, 23, 24] and seven were single-center studies [15–22]. The interventions of PS and T-Piece included in the meta-analysis are outlined in Table 2.

**Table 1:** The basic characteristics of studies included in meta-analysis

Author	Year	Country	Study period	Study design	No. of patients		
					Total	PS	T-piece
Ebsteban	1997	Spain	Oct. 1994–Jun. 1995	Multicenter	484	238	246
Koh	2000	Korea	May 1997–Mar. 1998	Single center	42	20	22
Haberthur	2002	Switzerland	Jul. 1997–Jul. 1998	Single center	60	30	30
Matic	2004	Croatia	Aug. 1999–Oct. 2000	Single center	260	150	110
Matic	2007	Croatia	Apr. 2004–Apr. 2006	Single center	136	70	66
Zhang	2014	China	Jan. 2007–Dec. 2007	Single center	208	93	115
Teixeira	2015	Brazil	Nov. 2012–Nov. 2013	Single center	112	46	66
Chittawatanarat	2018	Thailand	Jun. 2011–Nov. 2013	Single center	520	260	260
Santos Pellegrini	2018	Brazil	2012–2016	Multicenter	190	91	99
Subira	2019	Spain	Jan. 2016–Apr. 2017	Multicenter	1153	575	578

**Table 2:** Interventions of PS and T-piece included in the meta-analysis

Study	PS	T-piece
Esteban 1997 Koh 2000 Haberthur 2002 Matic 2004 Matic 2007	PS 7 cm H_2_O 2 h PS 15 cm H_2_O, decrease 3–5 cm H_2_O/h PS 5 cm H_2_O, PEEP 5 cm H_2_O 2 h PS 8 cmH_2_O 2 h PS 18 cm H_2_O, decrease 2–4 cm H_2_O/time to 5 cm H_2_O	T-piece 2 h T-piece 1 h T-piece 2 h T-piece 2 h T-piece 2 h
Zhang 2014	PS 5 cm/H_2_O, PEEP 5 cm H_2_O, FiO_2_ 30% 30 min	T-piece 4 L/min 30 min
Teixeira 2015 Chittawatanarat 2018 Santos Pellegrini 2018 Subira 2019	PS 7 cm H_2_O, PEEP 5–8 cm H_2_O, FiO_2_ ≤ 45% 30–90 min PS 5–7 cm H_2_O, PEEP 5 cm H_2_O, FiO_2_ 40% 120 min PS 10 cm H_2_O 30 min PS 8 cmH_2_O, PEEP 0 30 min	T-piece 30–90 min T-piece 10–15 L/min 120 min T-piece 30 min T-piece 2 h

### Primary outcome

**Fig. 4:**
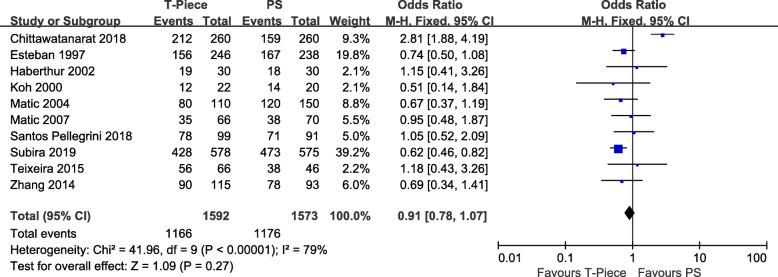
Forest plot for successful extubation rate

**Fig. 5:**
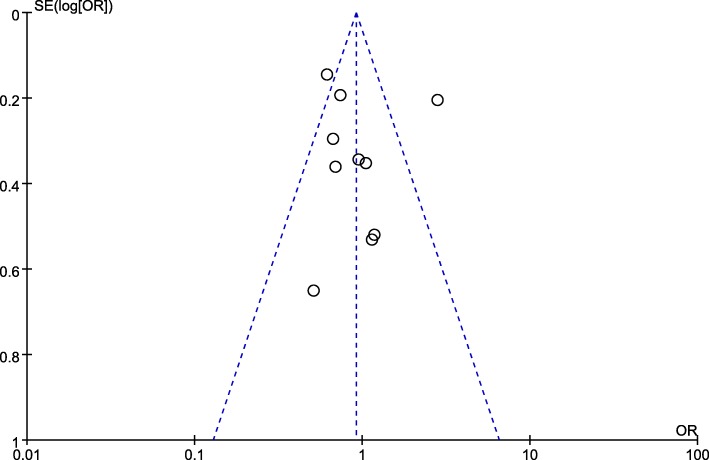
Funnel plot for successful extubation rate

A total of 10 RCTs including 3165 patients were included, and the successful extubation rate was about 74.0% (1166/1592 in the T-piece group and 1176/1573 in the PS group). There was no significant difference of successful extubation rate between T-piece group and PS group using the random effect model (odds ratio [OR] = 0.91;95% CI, 0.78–1.07; *P* = 0.27; chi^2^ = 41.96; *I*^2^ = 79%) (Fig. 4). A funnel plot was used to assess the publication bias (Fig. 5).

### Secondary outcomes

#### Rate of reintubation

**Fig. 6:**
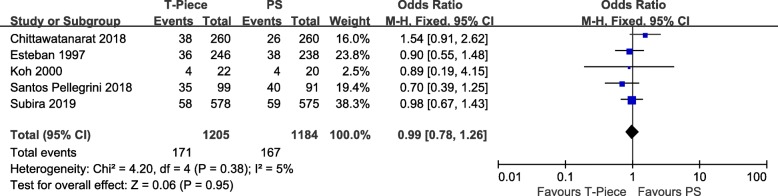
Forest plot for rate of reintubation

Five of included studies were analyzed to assess the rate of reintubation. The rate of reintubation was about 14.1% (171/1205 in the T-piece group and 167/1184 in the PS group). There was no statistically significant difference in the rate of reintubation between 2 groups (odds ratio [OR] = 0.99;95% CI, 0.78–1.26; *P* = 0.95; chi^2^ = 4.20; *I*^2^ = 5%) (Fig. 6).

#### ICU mortality

**Fig. 7:**
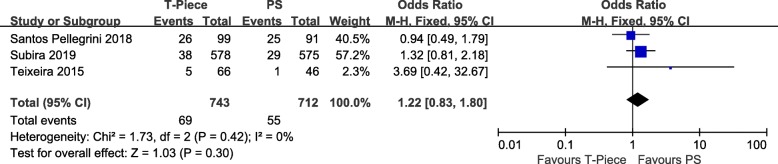
Forest plot for ICU mortality

Three of the included studies were analyzed to assess the ICU mortality. There was no statistically significant difference in the ICU mortality between 2 groups (odds ratio [OR] = 1.22; 95% CI, 0.83–1.80; *P* = 0.30; chi^2^ = 1.73; *I*^2^ = 0%) (Fig. 7).

#### Hospital mortality

**Fig. 8:**
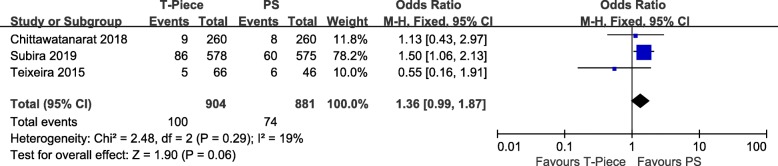
Forest plot for hospital mortality

Three of included studies were analyzed to assess the hospital mortality. There was no statistically significant difference in the hospital mortality between 2 groups (odds ratio [OR] = 1.36; 95% CI, 0.99–1.87; *P* = 0.06; chi^2^ = 2.48; *I*^2^ = 19%) (Fig. 8).

#### ICU length of stay

**Fig. 9:**
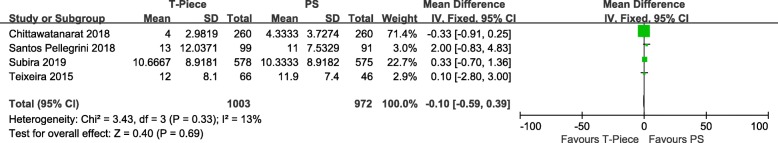
Forest plot for ICU length of stay

Four of included studies were analyzed to assess the ICU length of stay. There was no statistically significant difference in the ICU length of stay between 2 groups (Mean difference = − 0.10; 95% CI, − 0.59 to 0.39; *P* = 0.69; chi^2^ = 3.43; *I*^2^ = 13%) (Fig. 9).

#### Hospital length of stay

**Fig. 10:**
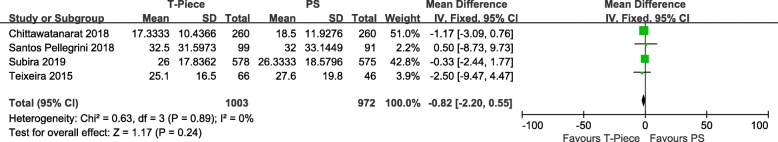
Forest plot for hospital length of stay

Four of included studies were analyzed to assess the hospital length of stay. There was no statistically significant difference in the hospital length of stay between 2 groups (Mean difference = − 0.82; 95% CI, − 2.2 to 0.55; *P* = 0.24; chi^2^ = 0.63; *I*^2^ = 0%) (Fig. 10).

## Discussion

This systematic review and meta-analysis of ten unique RCTs including 3165 patients compared T-piece and pressure support ventilation as spontaneous breathing trials in critically ill patients. We found that the overall successful extubation rate was about 74.0% and there was no significant difference of successful extubation rate between the T-piece group and PS group. Extubation failure may occur because of upper-airway obstruction, ineffective cough, and excessive respiratory secretions that cannot be managed by the patient [2]. Another potential reason for extubation failure is loss of positive pressure in the chest after extubation in subjects weaned to PSV [25]. PSV allows patients to retain control over respiratory rate and timing, inspiratory flow rate, and tidal volume. In addition, physicians can modulate a satisfactory workload for the patients by monitoring breathing frequency and accessory muscle activity during PSV. Because of these potential advantages, the value of PSV as a technique to gradually withdraw ventilator support is generally recognized for patients who have weaning difficulties [17]. Sklar et al. [3] recently pointed out that PSV significantly reduces the work of breathing and pressure-time product compared to the T-piece, which could, in turn, more closely represent the post-extubation scenario. However, noninvasive mechanical ventilation (NIV) dissemination as an adjunctive for extubation makes clinical interpretation of these data difficult [24]. The major finding of our study suggests that both spontaneous breathing using T-piece and PSV are suitable methods for successful extubation of patients with critical illness from mechanical ventilation.

The main goal of a weaning trial is to identify patients who are able to breathe without a ventilator with the minimum risk of extubation failure and its potential complications [26]. Daily screening of respiratory function by SBT is associated with a shorter duration of mechanical ventilation [27]. After a successful SBT and extubation, 10 to 25% of patients require reintubation, and reintubation is associated with higher mortality [28, 29]. In this meta-analysis, the reintubation rate was not significantly different between the 2 groups (about 14.1%), which is lower than the 17% in the first study by Esteban et al. [16] and similar to the 13% in their second study [30]. Conversely, the reintubation rate was higher than in a study by Perren et al. [31] (9% for short SBTs and 4% for long SBTs), but that study has a single-center design and the small sample size precludes direct comparison.

Hospital mortality and ICU mortality were not statistically significant different between 2 groups. ICU or hospital mortality may be not directly related to the SBT technique which is the intervention that is applied for a very short period during the course of ICU admission. Patient mortality is associated with prolonged intubation or unsuccessful weaning and they significantly increased medical costs because of extended hospitalization. Besides this, we also found that hospital length of stay and ICU length of stay were not statistically significant different between 2 groups. This finding can be explained by the reintubation rate, APACHE II score at admission, and the overall successful extubation rate, which were not significantly different between the 2 groups.

A variety of workers have indicated that continuous positive airway pressure of 5 cm H_2_O, typically considered as minimal support, decreases patient work of breathing by as much as 40%. Pressure support of 5 cm H_2_O also decreases patient work of breathing by 30 to 40% [32, 33]. The vast majority of patients can cope with a 40 to 60% increase in work of breathing at the point of extubation, but a fragile patient may not [34]. The small population of marginal patients will likely require reintubation. Reintubation is associated with a significant mortality rate. It is necessary to look for the high-risk patient and treat all patients as vulnerable and assess their ability to breathe.

Our meta-analysis has several characteristics: (1) We conducted a systematic search of several databases to identify all RCTs comparing T-piece and PSV SBT techniques in weaning subjects. (2) We employed standardized techniques to assess risk of bias and overall quality of evidence.

This meta-analysis is associated with several limitations. First, the number of included studies is small. Further randomized clinical trials should be conducted in order to assess whether or not PSV is safer and more effective compared to the T-piece method for achieving relevant clinical outcomes among adult patients with at least 24 h of invasive ventilation. Second, many of the secondary outcomes such as hospital length of stay or hospital mortality were not included in all of the studies examined in this meta-analysis. Third, the rate of SBT success is also very important because successful extubation after passing the SBT will be also related to upper airway patency and adequacy of secretion clearance. Only if the patients have the above conditions can they pass the SBTs. However, not all of included studies showed this data. Fourth, there was substantial heterogeneity among the included studies. Therefore, our findings should be interpreted with caution.

## Conclusion

T-piece and PSV as SBTs are considered to have comparable predictive power of successful extubation in critically ill patients. The analysis of secondary outcomes also shows no significant difference in the rate of reintubation, ICU and hospital length of stay, and ICU and hospital mortality between the 2 groups. Further randomized controlled studies of SBTs are still required.

## Abbreviations

CI: Confidence interval
ICU: Intensive care unit
MD: Mean difference
NIV: Noninvasive mechanical ventilation
OR: Risk ratio
PRISMA: Preferred Reporting Items for Systematic Reviews and Meta-Analyses
PSV: Pressure support ventilation
RCTs: Randomized controlled trials
SBT: Spontaneous breathing trial
